# Healthcare-associated *Stenotrophomonas maltophilia* infections in the United States, 2018–2022

**DOI:** 10.1017/ash.2023.352

**Published:** 2023-09-29

**Authors:** Amelia Keaton, Lucy Fike, Kevin Spicer, Alexander Kallen, Kiran Perkins

## Abstract

**Background:** *Stenotrophomonas maltophilia* is an important cause of opportunistic healthcare-associated infections (HAIs) in critically ill patients and is difficult to treat due to intrinsic resistance to multiple antibiotic classes. During the COVID-19 pandemic, the CDC received anecdotal reports of increases in *S. maltophilia* respiratory infections. To further investigate these reports, we used a national electronic healthcare database to evaluate changes in *S. maltophilia* during the pandemic. **Methods:** Using the PINC-AI healthcare data (Premier Inc, Charlotte, NC) we identified all potential HAIs by calculating the total number of unique patients hospitalized during January 1, 2018, through December 31, 2021, who had any organism isolated on clinical culture obtained >3 days after admission. We calculated the proportion of patients with *S. maltophilia* detected in culture and stratified them by specimen source. To determine whether COVID-19 diagnosis influenced the proportion of patients diagnosed with *S. maltophilia* respiratory infections during the pandemic (January 1, 2020–December 31, 2021), we calculated the proportion of patients with *S. maltophilia* detected among those with any bacterial pathogen isolated from a respiratory culture >3 days after hospitalization. We stratified these results by presence or absence of concurrent COVID-19 diagnosis. Pearson χ^2^ test was used to test for differences where appropriate. **Results:** Among hospitalized patients with any organism isolated from a clinical culture, the proportion with *S. maltophilia* detected was higher in 2021 (n = 2,554 of 118,029, 2.2%) than in 2018 (n = 2,063 of 155,624, 1.3%) p 3 days after hospital admission from 2018 to 2021. Most patient isolates were from respiratory specimens. A concurrent diagnosis of COVID-19 did not appear to increase the likelihood of respiratory *S. maltophilia* detection. The increases in *S. maltophilia* during the pandemic might be explained by challenges inherent to caring for increased numbers of higher-acuity patients during this time, including staffing shortages and changes to infection prevention practices. Additional exploration of these data, as well as data from other sources and from additional years, may help to elucidate this issue more fully.

**Disclosures:** None

